# Patient selection, practice patterns, and perioperative outcomes for robotic splenectomy: a contemporary ACS-NSQIP analysis

**DOI:** 10.1007/s00464-026-12964-6

**Published:** 2026-06-09

**Authors:** Mohammad Saad Farooq, Anish Jona, Gracia Maria Vargas, Russell A. Simons, John T. Miura, Giorgos C. Karakousis, Mark S. Etherington

**Affiliations:** Division of Endocrine and Oncologic Surgery, Hospital of the University of Pennsylvania, 3400 Spruce Street, 4 Maloney, Philadelphia, PA 19104 USA

**Keywords:** Splenectomy, Robotic, Laparoscopic, Outcomes, NSQIP, Robotic surgery

## Abstract

**Background:**

Robot-assisted surgery has increasingly been adopted across multiple surgical specialties, demonstrating benefits such as enhanced precision and visualization, and potentially improved outcomes. However, evidence for its use in less common operations, including splenectomy, remains limited. To better understand its use and outcomes, we examined practice patterns and perioperative results of robotic splenectomy in a large, contemporary national cohort.

**Methods:**

The American College of Surgeons – National Surgical Quality Improvement Program database was queried for adult patients who underwent open, laparoscopic, or robotic splenectomy from 2022 to 2023; patient characteristics were categorized by type of surgical approach. Perioperative outcomes for robotic vs. laparoscopic splenectomy were analyzed using univariable and multivariable analyses.

**Results:**

Among 852 eligible patients that underwent splenectomy, 163 (19%) operations were performed using the robotic approach, while 339 (39.7%) were laparoscopic and 350 (41.1%) were open. The most common indications for robotic splenectomy were benign hematologic conditions (33%), neoplasm/mass/cyst (32%), and splenomegaly (18%).

Compared to laparoscopic surgery, robotic splenectomy was associated with longer operative time (152 vs. 130 min, *p* < 0.001) but shorter length of stay (3.6 vs. 5.2 days, *p* = 0.010). Notably, there were no significant differences in the proportion of patients experiencing an unplanned conversion to open surgery, postoperative blood transfusions, or other postoperative complications between the robotic and laparoscopic cohorts.

**Conclusion:**

In this large contemporary national cohort, robotic splenectomy was safe, with low morbidity, shorter hospitalization, and longer operative times compared to laparoscopic surgery. These findings support the feasibility and safety of robotic splenectomy with appropriate patient selection.

Robot-assisted surgery has been increasingly utilized over the past decade, with multiple surgical disciplines instituting the robotic approach due to similar or potentially improved perioperative outcomes [1]. While data on robotic outcomes for multiple surgical subspecialties have recently become available, there remain gaps in the literature for less commonly performed operations, such as splenectomy, for which the surgical robot may offer technical advantages. These include the ability to more precisely dissect vessels in the splenic hilum, and to more reliably identify the tail of the pancreas [2].

Though minimally invasive surgery has been widely utilized for elective splenectomies with potentially improved outcomes compared to open surgery [3], data on robotic splenectomy are limited to small, single-institution cohorts and case studies with mixed findings regarding operative time, blood loss, conversions to open, and perioperative outcomes [2, 4–9]. Two recent systematic reviews utilizing the available literature illustrate this uncertainty: Bhattacharya et al. reported reduced intraoperative blood loss with robotic splenectomy; however, no differences in operative time, length of stay, or postoperative outcomes were found [10]. Conversely, Madan et al. reported lower conversion rates and improved perioperative outcomes with the robotic approach [11].

Notably, the aforementioned summarized literature includes heterogeneous studies, encompassing patients from a variety of countries, healthcare systems, and time periods that span up to 20 years ago [2, 4–8, 12, 13]; additionally, studies with both pediatric and adult populations are included, as well as partial and total splenectomies. Variations in reporting of patient factors, surgical indications, and perioperative outcomes further limit the generalizability of existing data [6, 10, 11, 14].

Given the increasing adoption of robotic splenectomy, a comprehensive and contemporary analysis is warranted. Using the National Surgical Quality Improvement Program (NSQIP) database, we characterized practice patterns, patient factors, and perioperative outcomes of robotic splenectomy in adults, and compared these outcomes with the laparoscopic approach in a modern cohort. We hypothesized a priori that robotic splenectomy would have comparable outcomes to laparoscopic splenectomy.

## Methods

### Study population

**Fig. 1:**
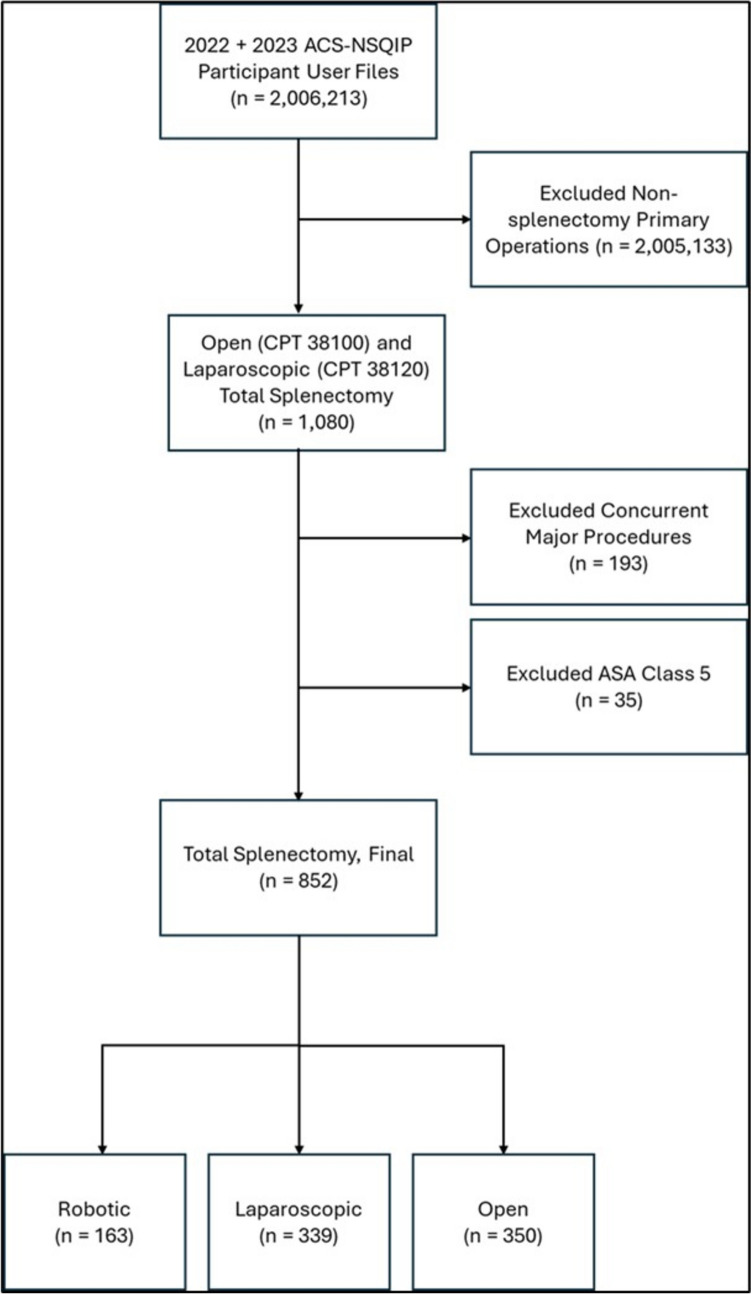
Flowchart of inclusion criteria

This was a retrospective national cohort observational study. The American College of Surgeons NSQIP database, a nationally validated, risk-adjusted database tracking surgical outcomes [15], was queried for adult patients (> 18 years of age) who underwent open total splenectomy (common procedural terminology [CPT] codes: 38100) or laparoscopic total splenectomy (CPT codes: 38120). The year of surgery was limited to 2022 and 2023, as these were the first years in which a specific “robot_used” variable was added to the general NSQIP participant user file, allowing granular identification of patients who underwent robot-assisted splenectomy. Patients who underwent any concurrent major surgery apart from splenectomy were strictly excluded in order to minimize heterogeneity and enable a more direct comparison between surgical approaches. Additionally, patients classified as American Society of Anesthesiologists (ASA) class 5 (i.e., moribund) were excluded. Patients were stratified based on their intended initial surgery, regardless of whether it resulted in an unplanned conversion to an open procedure. The overall cohort inclusion criteria are summarized in Fig. 1.

### Clinicopathologic variables

Specific patient variables collected included age, sex, race, ethnicity, ASA class, body mass index (BMI), smoking status, presence of comorbidities (diabetes mellitus, chronic obstructive pulmonary disease [COPD], congestive heart failure [CHF]), case type (elective vs. urgent/emergent), inpatient status, and indication for surgery based on the International Classification of Diseases, 10th Revision. 

### Outcome measures

Specific outcome measures collected included operative time, length of stay (LOS), unplanned conversion to open surgery, unplanned readmission rate, 30-day mortality rate, and 30-day postoperative complication rates: skin/soft tissue infection (SSI), deep/organ space infection, urinary tract infection (UTI), pneumonia, sepsis, deep vein thrombosis, pulmonary embolism, blood transfusion, unplanned return to the operating room (OR), unplanned intubation, renal failure, and cardiac arrest.

### Statistical analyses

The study population was presented using descriptive statistics, with medians with interquartile ranges (IQR) and/or means with standard deviations reported for continuous variables. Continuous variables were analyzed using Wilcoxon rank-sum tests and unpaired t-tests, while categorical variables were analyzed with Pearson’s chi-squared tests and Fisher’s exact tests as appropriate. Univariable analysis was conducted to characterize factors associated with undergoing the robotic approach in the overall cohort vs. laparoscopic and open approaches.

Multivariable Firth’s logistic regression was used to assess factors associated with undergoing robotic surgery vs. laparoscopic surgery, with results reported as adjusted odds ratios (aOR) with 95% confidence intervals (CI). Individual postoperative outcomes were compared between robotic and laparoscopic splenectomies with univariable analysis. A subset analysis of postoperative outcomes for elective cases only was also conducted. Post hoc multivariable Firth’s logistic regression analyses of factors associated with select postoperative outcomes (> 75th percentile LOS and operative time) were conducted to test the robustness of findings.

All analyses were conducted using Stata software (StataCorp, version 17). A *p* value of < 0.05 was utilized to denote statistical significance. This study was exempt from Institutional Review Board approval as it used deidentified patient information from a national database.

## Results

### Patient, disease, and surgical characteristics

Of 852 eligible patients that underwent splenectomy, 163 (19.1%) operations were performed using the robotic approach, while 339 (39.7%) were laparoscopic and 350 (41.1%) were open. Patients who underwent robotic splenectomy had a median age of 53 years (IQR: 37–66) and were predominantly female (63%) and white (66%). The most common indications for robotic splenectomy were benign hematologic conditions (33%), neoplasm/mass/cyst (32%), and splenomegaly (18%; Table 1).

**Table 1:** Clinicopathologic factors of overall patient cohort stratified by surgical approach with univariable analysis

Factor	Robotic (*n* = 163)	Laparoscopic (*n* = 339)	*p* value (robotic vs. lap)	Open (*n* = 350)	*p* value (robotic vs. open)
Age at diagnosis (yrs), median (IQR)	53 (37–66)	53 (35–66)	0.997	57.5 (43–69)	**0.004**
Sex, *n* (%)			**0.015**		**0.005**
Male	60 (36.8)	164 (48.4)		175 (50.0)	
Female	103 (63.2)	175 (51.6)		175 (50.0)	
Non-binary	0 (0)	0 (0)		0 (0)	
Race/ethnicity, *n* (%)			** < 0.001**		** < 0.001**
White	107 (65.6)	171 (50.4)		226 (64.6)	
Black	19 (11.7)	24 (7.1)		33 (9.4)	
Hispanic	22 (13.5)	26 (7.7)		13 (3.7)	
Asian/Pacific Islander	7 (4.3)	9 (2.7)		3 (0.9)	
Other/unreported	8 (4.9)	109 (32.2)		75 (21.4)	
Inpatient status, *n* (%)	124 (76.1)	256 (75.5)	0.892	342 (97.7)	** < 0.001**
ASA class, *n* (%)			0.148		** < 0.001**
1	2 (1.2)	8 (2.4)		1 (0.3)	
2	58 (35.6)	113 (33.3)		53 (15.1)	
3	95 (58.3)	182 (53.7)		186 (53.1)	
4	8 (4.9)	36 (10.6)		110 (31.4)	
BMI, median (IQR)	28.1 (24.0–33.5)	28.3 (24.4–32.7)	0.881	26.3 (22.1–31.1)	**0.001**
Smoking status, *n* (%)			0.823		** < 0.001**
No	143 (87.7)	295 (87.0)		256 (73.1)	
Yes	20 (12.3)	44 (13.0)		94 (26.9)	
Diabetes, *n* (%)			0.416		0.913
No	138 (84.7)	296 (87.3)		295 (84.3)	
Yes	25 (15.3)	43 (12.7)		55 (15.7)	
COPD, *n* (%)			0.765		0.072
No	158 (96.9)	331 (97.6)		324 (92.6)	
Yes	5 (3.1)	8 (2.4)		26 (7.4)	
CHF, *n* (%)			0.094		**0.018**
No	160 (98.2)	321 (94.7)		326 (92.6)	
Yes	3 (1.8)	18 (5.3)		24 (6.9)	
Indication/pathology, *n* (%)			0.255		** < 0.001**
Benign hematologic	53 (32.5)	133 (39.2)		21 (6.0)	
Neoplasm/mass/cyst	52 (31.9)	88 (26.0)		47 (13.4)	
Splenomegaly	29 (17.8)	48 (14.2)		59 (16.9)	
Vascular aneurysm/thrombosis/infarct	8 (4.9)	26 (7.7)		90 (25.7)	
Infection/abscess	4 (2.5)	5 (1.5)		23 (6.6)	
Trauma/hemorrhage	0 (0)	5 (1.5)		87 (24.9)	
Other	17 (10.4)	34 (10.0)		23 (6.6)	
Case type, *n* (%)			0.050		** < 0.001**
Elective	154 (94.5)	302(89.1)		162 (46.3)	
Urgent/emergent	9 (5.5)	37 (10.9)		188 (53.7)	
Hand port utilized, *n* (%)			**0.005**		–
No	149 (91.4)	277 (81.7)		–	
Yes	14 (8.6)	62 (18.3)		–	

Bold represents *p* value < 0.05

On univariable analysis, compared to laparoscopic splenectomy, patients who underwent robotic splenectomy were more likely to be female (63 vs. 52%, *p* = 0.015) and undergo an elective operation (95 vs. 89%, *p* = 0.05). Additionally, patients who underwent robotic splenectomy were more likely to be white (66 vs. 50%, *p* < 0.001), though this field was not reported for 32% of patients who underwent laparoscopic splenectomy. Lastly, patients who underwent robotic splenectomy were less likely to have a hand-assist port utilized during surgery (8.6 vs. 18.3%, *p* = 0.005). The remaining clinicopathologic and surgical factors did not significantly differ between the robotic and laparoscopic cohorts (Table 1).

When comparing robotic splenectomy with open splenectomy, there were significant differences in clinicopathologic characteristics. Patients in the open cohort were older (57.5 vs. 53 years, *p* = 0.004), more likely to be male (50 vs. 37%, *p* = 0.005), had a higher ASA class (31 vs. 5% class 4, *p* < 0.001), were designated as inpatients (98 vs. 76%, *p* < 0.001), were active smokers (27 vs. 12%, *p* < 0.001), and had CHF (7 vs. 2%, *p* = 0.018). Most notably, the open cohort was much more likely to undergo urgent/emergent surgery (54 vs. 6%, *p* < 0.001) and coincidingly more often underwent surgery for trauma/hemorrhage (25 vs. 0%, *p* < 0.001) and vascular aneurysm/thrombosis (26 vs. 5%, *p* < 0.001; Table 1). Given the stark differences in patient populations, the subsequent outcomes analyses were conducted between the robotic and laparoscopic cohorts only.

### Factors associated with undergoing robotic vs. laparoscopic splenectomy on multivariable analysis

On multivariable logistic regression analysis, female patients were more likely to undergo robotic surgery vs. laparoscopic (aOR: 1.62, 95% CI 1.08–2.41, *p* = 0.019). Patients undergoing surgery for neoplasm/mass also trended toward higher likelihood of undergoing robotic surgery (aOR: 1.51, 95% CI 0.93–2.44, *p* = 0.097; Table 2).

**Table 2:** Multivariable regression of factors associated with undergoing robotic splenectomy (vs. laparoscopic)

Factor	Adjusted odds ratio	95% confidence interval	*p*-value
Age at diagnosis	1.00	0.99–1.01	0.912
Sex			
Male	1 (ref)		
Female	1.62	1.08–2.41	**0.019**
ASA class			
1	1 (ref)		
2	2.07	0.47–9.03	0.335
3	2.08	0.48–9.06	0.332
4	1.08	0.21–5.64	0.924
Case classification			
Elective	1 (ref)		
Urgent/emergent	0.80	0.35–1.81	0.589
Inpatient status			
No	1 (ref)		
Yes	0.85	0.54–1.34	0.490
BMI	0.99	0.97–1.02	0.756
Smoking status			
No	1 (ref)		
Yes	1.08	0.60–1.95	0.797
Diabetes			
No	1 (ref)		
Yes	1.44	0.80–2.58	0.220
COPD			
No	1 (ref)		
Yes	1.77	0.54–5.81	0.346
CHF			
No	1 (ref)		
Yes	0.46	0.14–1.56	0.214
Indication/pathology			
Benign hematologic	1 (ref)		
Neoplasm/mass/cyst	1.51	0.93–2.44	0.097
Splenomegaly	1.47	0.83–2.61	0.183
Vascular aneurysm/thrombosis/infarct	0.72	0.30–1.70	0.448
Infection/abscess	2.95	0.76–11.42	0.117
Trauma/hemorrhage	0.32	0.02–6.20	0.452
Other	1.24	0.63–2.41	0.535

Bold represents *p* value < 0.05

### Individual perioperative outcomes for robotic vs. laparoscopic splenectomy

Robotic surgery was associated with a shorter LOS vs. the laparoscopic approach: mean 3.6 vs. 5.2 days (*p* = 0.010) and median 2 (IQR: 1–3) vs. 2 days (IQR: 1–5). The robotic approach also exhibited longer operative time (152 vs. 130 min, *p* < 0.001). Notably, there were no significant differences in the proportion of patients experiencing an unplanned conversion to open surgery (3.1 vs. 2.9%, *p* > 0.900), postoperative blood transfusions (7.4 vs. 10.3%, *p* = 0.286), or other postoperative complications between the robotic and laparoscopic cohorts (Table 3).

**Table 3:** Univariable analysis of perioperative outcomes for patients undergoing robotic vs. laparoscopic splenectomy (overall cohort)

Outcome	Robotic (*n* = 163)	Laparoscopic (*n* = 339)	*p* value
Length of stay (days)			**0.010**
Median (IQR)	2 (1–3)	2 (1–5)	
Mean (SD)	3.6 (5.5)	5.2 (7.6)	
Operative time (minutes), median (IQR)	152 (119–195)	130 (93–172)	** < 0.001**
Unplanned conversion to open, *n* (%)	5 (3.1)	10 (2.9)	> 0.900
Readmission, *n* (%)	18 (11.0)	29 (8.6)	0.370
30-day mortality, *n* (%)	0 (0.0)	2 (0.6)	> 0.900
Post-op blood transfusion, *n* (%)	12 (7.4)	35 (10.3)	0.286
Skin/soft tissue infection, *n* (%)	0 (0.0)	0 (0.0)	**–**
Deep/organ space infection, *n* (%)	1 (0.6)	7 (2.1)	0.447
Urinary tract infection, *n* (%)	0 (0.0)	1 (0.3)	> 0.900
Pneumonia, *n* (%)	3 (1.8)	8 (2.4)	> 0.900
Sepsis, *n* (%)	1 (0.6)	1 (0.3)	0.544
Deep vein thrombosis, *n* (%)	7 (4.3)	17 (5.0)	0.826
Pulmonary embolism, *n* (%)	0 (0.0)	3 (0.9)	0.554
Renal failure, *n* (%)	1 (0.6)	3 (0.9)	> 0.900
Cardiac arrest, *n* (%)	1 (0.6)	0 (0.0)	0.325
Unplanned intubation, *n* (%)	1 (0.6)	3 (0.9)	> 0.900
Reoperation, *n* (%)	5 (3.1)	11 (3.2)	> 0.900

Bold represents *p* value < 0.05

### Multivariable logistic regression of factors associated with prolonged length of stay and operative time

Post hoc assessment of factors associated with > 75th percentile LOS in the laparoscopic/robotic cohort (4 days) was conducted. Undergoing robotic surgery was significantly associated with reduced likelihood of prolonged LOS (aOR: 0.48, 95% CI 0.28–0.82, *p* = 0.008). Urgent/emergent cases were highly associated with increased likelihood of prolonged LOS (aOR: 10.45, 95% CI 4.49–24.33, *p* < 0.001; Table 4).

**Table 4:** Multivariable logistic regression of factors associated with > 75th percentile length of stay (4 days) and operative time (181 min)

	Length of stay > 4 days			Operative time > 181 min		
Factor	adjusted odds ratio	95% confidence interval	*p* value	adjusted odds ratio	95% confidence interval	*p* value
Surgery type						
Laparoscopic	1 (ref)			1 (ref)		
Robotic	0.48	0.28–0.82	**0.008**	1.64	1.07–2.53	**0.024**
Age at diagnosis	1.00	0.99–1.02	0.863	0.99	0.98–1.00	0.057
Sex						
Male	1 (ref)			1 (ref)		
Female	0.78	0.48–1.26	0.306	0.68	0.44–1.04	0.077
ASA class						
1	1 (ref)			1 (ref)		
2	0.38	0.08–1.94	0.247	5.94	0.33–106.35	0.226
3	1.03	0.21–5.11	0.973	8.52	0.47–153.48	0.146
4	2.07	0.37–11.63	0.410	10.20	0.52–198.73	0.125
Case classification						
Elective	1 (ref)			1 (ref)		
Urgent/emergent	10.45	4.49–24.33	** < 0.001**	0.82	0.37–1.84	0.634
BMI	0.97	0.95–1.01	0.104	0.99	0.96–1.01	0.265
Indication/pathology						
Benign hematologic	1 (ref)			1 (ref)		
Neoplasm/mass/cyst	0.67	0.36–1.26	0.217	1.12	0.65–1.93	0.685
Splenomegaly	1.06	0.54–2.09	0.870	1.93	1.07–3.49	**0.029**
Vascular aneurysm/thrombosis/infarct	1.52	0.61–3.78	0.371	1.41	0.60–3.29	0.430
Infection/abscess	1.88	0.39–8.95	0.429	0.92	0.20–4.14	0.910
Trauma/hemorrhage	3.58	0.29–43.73	0.318	1.25	0.18–8.78	0.821
Other	1.22	0.56–2.66	0.617	1.09	0.52–2.27	0.813

Bold represents *p* value < 0.05

We additionally assessed factors associated with > 75th percentile operative time in the laparoscopic/robotic cohort (181 min). Robotic surgery was associated with increased likelihood of prolonged operative time (aOR: 1.64, 95% CI 1.07–2.53, *p* = 0.024). Additionally, surgeries conducted for splenomegaly were also more likely to have prolonged operative time (aOR: 1.93, 95% CI 1.07–3.49, *p* = 0.029; Table 4).

### Subset analysis of elective cases only

We conducted a subset analysis after removal of urgent/emergent cases, as this factor was highly associated with prolonged LOS. There remained 154 patients in the robotic cohort and 302 in the laparoscopic cohort. Similar to the overall cohort analysis, robotic surgery was associated with a shorter length of stay and increased operative time vs. laparoscopic, but there were no differences in the remaining perioperative outcomes (Table 5).

**Table 5:** Univariable analysis of perioperative outcomes for patients undergoing robotic vs. laparoscopic splenectomy (subset analysis of elective cases only)

Outcome	Robotic (*n* = 154)	Laparoscopic (*n* = 302)	*p* value
Length of stay (days)			**0.028**
Median (IQR)	2 (1–3)	2 (1–4)	
Mean (SD)	2.8 (3.6)	4.3 (6.5)	
Operative time (minutes), median (IQR)	150.5 (118–194)	132 (93–174)	** < 0.001**
Unplanned conversion to open, *n* (%)	4 (2.6)	8 (2.6)	> 0.900
Readmission, *n* (%)	17 (11.0)	25 (8.3)	0.335
30-day mortality, *n* (%)	0 (0.0)	1 (0.3)	> 0.900
Post-op blood transfusion, *n* (%)	11 (7.1)	30 (9.9)	0.324
Skin/soft tissue infection, *n* (%)	0 (0.0)	0 (0.0)	**–**
Deep/organ space infection, *n* (%)	1 (0.6)	6 (2.0)	0.432
Urinary tract infection, *n* (%)	0 (0.0)	0 (0.0)	**–**
Pneumonia, *n* (%)	3 (1.9)	5 (1.7)	> 0.900
Sepsis, *n* (%)	1 (0.6)	1 (0.3)	> 0.900
Deep vein thrombosis, *n* (%)	6 (3.9)	17 (5.6)	0.503
Pulmonary embolism, *n* (%)	0 (0.0)	3 (1.0)	0.554
Renal failure, *n* (%)	1 (0.6)	4 (1.3)	0.667
Cardiac arrest, *n* (%)	1 (0.6)	0 (0.0)	0.338
Unplanned intubation, *n* (%)	1 (0.6)	2 (0.7)	> 0.900
Reoperation, *n* (%)	5 (3.2)	10 (3.3)	> 0.900

Bold represents *p* value < 0.05

## Discussion

In this NSQIP analysis of patients undergoing robotic splenectomy, we found that when compared to laparoscopic splenectomy, the robotic approach was associated with a shorter LOS and a longer operative time. Additionally, there were minor differences in patient characteristics and indications for surgery when comparing the robotic and laparoscopic approaches. To our knowledge, this is the first analysis to highlight practice patterns and perioperative outcomes of robotic splenectomy using the NSQIP database, with the largest cohort size from an individual study thus far in the literature.

Our findings specifically emphasize that robotic splenectomy is safe, with comparable perioperative morbidity with the laparoscopic approach. Prior studies have reported reduced volume of blood loss with robotic splenectomy [4, 10] however, we did not find a difference in the rate of postoperative blood transfusion between the robotic vs. laparoscopic cohorts. Notably, the NSQIP database does not provide data on intraoperative blood loss, limiting direct comparison with prior studies reporting this metric. Importantly, there were no significant differences in the remaining postoperative outcomes between the robotic and laparoscopic cohorts, including conversions to open surgery, with both groups exhibiting low overall morbidity.

These findings are reassuringly consistent with some prior single-institution analyses. Notably, however, compared to prior studies, LOS in our minimally invasive cohort (robotic and laparoscopic) was shorter (2 days vs. 6–7 days) [11]. This may be due to heterogeneity in studies utilized for prior summary analyses, whereas we utilized a more contemporary cohort from centers across North America.

In our analysis, there were stark differences between patients who underwent robotic surgery vs. open surgery, with the open cohort comprised of older, more comorbid patients, who more often underwent emergent surgery and surgery for trauma/hemorrhage. Conversely, patients with benign hematologic conditions or neoplasms were more likely to undergo a minimally invasive approach. This finding is unsurprising, but questions remain regarding whether the robot affords additional exposure to minimally invasive approaches for patients who would otherwise undergo an open operation, or whether this decision hinges upon surgeon preference/experience and available resources.

Additionally, given the reported higher costs and increased operative times associated with robotic surgery [16], the cost-effectiveness of this approach should be evaluated on a case-by-case basis, considering patient factors, indications for surgery, and surgeon expertise. The increased operative time may reflect docking and setup time or early adoption learning curves. Despite this, the reduction in LOS may partially mitigate the impact of prolonged operative time, particularly in well-selected elective cases, as has been demonstrated for other general surgery operations [17, 18]. To further clarify whether these differences were attributable to the robotic platform itself or to other patient- or surgery-related factors, we performed multivariable analyses examining predictors of prolonged LOS and operative time. After adjustment, the robotic approach remained independently associated with a lower likelihood of prolonged LOS and a higher likelihood of prolonged operative time. These findings suggest that the observed reduction in LOS persists beyond measured confounders, although residual confounding and differences in patient selection or perioperative management may still contribute.

As robotic surgery becomes more widely adopted, further studies are warranted to evaluate long-term outcomes, cost-effectiveness, and the potential role of the robotic approach in expanding minimally invasive surgery to higher-risk or more complex splenectomy cases.

There are certain limitations to this study, notably its retrospective design limited to 2 years of NSQIP data. Trends over time using the NSQIP database may offer further insight on robotic splenectomy practice patterns/outcomes. We also do not know details on potentially complicating surgical/anatomic considerations, such as the size of the spleen or a history of prior surgery; nevertheless, it is unlikely that this would differ between the robotic and laparoscopic approaches. Additionally, while this is a national cohort of patients, it is limited to centers that report to NSQIP, and is thus subject to inherent limitations, including potential selection bias from voluntary hospital participation, systematic case sampling, and the possibility of miscoding or unmeasured confounding despite standardized data collection and auditing processes, which may limit the generalizability of our findings.

## Conclusion

In this large, contemporary national cohort of adult patients undergoing splenectomy, the robotic approach was associated with a shorter LOS and longer operative time compared to laparoscopic surgery, while maintaining similarly low rates of postoperative complications. These findings demonstrate that robotic splenectomy is a safe and feasible minimally invasive option when patients are appropriately selected.
